# Correlation Between Interest in COVID-19 Hair Loss and COVID-19 Surges: Analysis of Google Trends

**DOI:** 10.2196/37271

**Published:** 2022-04-27

**Authors:** Joseph Han, Samir Kamat, Aneesh Agarwal, Ross O'Hagan, Connor Tukel, Shayan Owji, Sabrina Ghalili, Yen Luu, Cula Dautriche Svidzinski, Brian J Abittan, Jonathan Ungar, Nicholas Gulati

**Affiliations:** 1 Department of Dermatology Icahn School of Medicine at Mount Sinai New York, NY United States; 2 Department of Medical Education Icahn School of Medicine at Mount Sinai New York, NY United States; 3 School of Medicine University of Missouri Kansas City, MO United States

**Keywords:** COVID-19, SARS-CoV-2 virus, pandemic, hair loss, telogen effluvium, Google Trends, omicron, omicron variant, delta variant, public interest, stress, dermatology, public perception, social media, online health, digital dermatology

## Introduction

There is an increasing body of evidence documenting an appreciable incidence of hair loss in patients with a past history of COVID-19 [1]. In general, COVID-19 has been accompanied by reports of increased mental health stress; this has serious implications for the psychosocial well-being of the overall population given the most recent emergence and surge of the omicron variant, characterized by unprecedented infectivity and spread [2,3]. Since both stress and infection are potential factors leading to telogen effluvium (hair shedding), it is important to understand how the surges of the initial COVID-19 strain and subsequent variant strains have influenced public interest in telogen effluvium and hair loss [1].

## Methods

To assess the public perception between hair loss and COVID-19, we analyzed search volume data on the Google search engine for the terms “COVID hair loss” and “Telogen Effluvium,” using the Google Trends data set spanning from January 1, 2020, to January 16, 2022. New case counts for COVID-19 were obtained from a publicly available COVID-19 repository [4]. We associated average daily new cases with the number of Google Trends search results for “COVID hair loss” for each week using a linear regression model and also performed a Spearman rank correlation test. The Mann-Kendall test was used to determine the significance of the upward trend of search term data for “COVID hair loss” over time.

## Results

**Figure 1:**
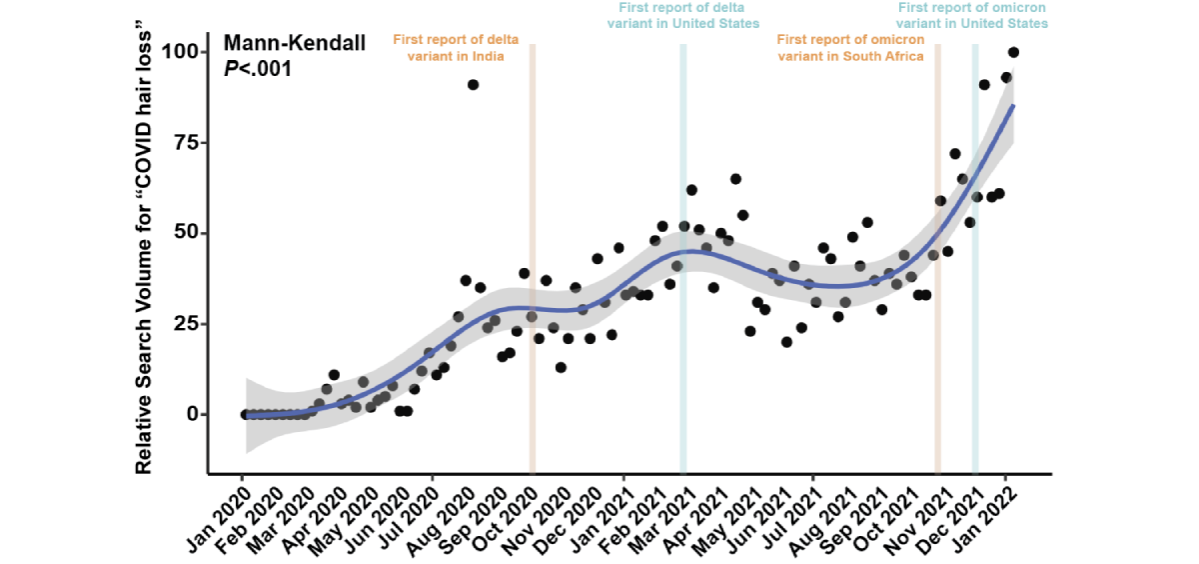
Trend over time of relative search volume results for “COVID hair loss” in the United States. The CI is shown in gray. The *P* value was determined using the Mann-Kendall test.

**Figure 2:**
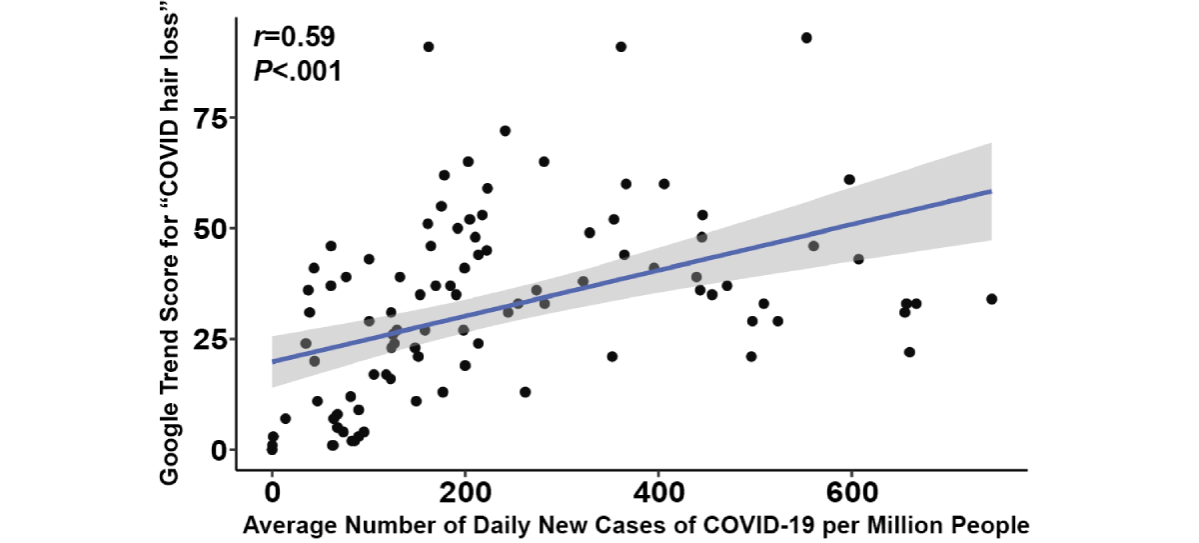
Spearman correlation between the relative search volume for “COVID hair loss” and the average number of daily new cases of COVID-19 in the United States per 1 million people. The *P* value was calculated using a linear regression model.

The relative search volume (RSV) for “COVID hair loss” significantly increased over time (*P*<.001) (Figure 1). The RSV for “COVID hair loss” first peaked during the initial surge in August 2020, and had local maxima during the subsequent delta and omicron variant surges, with an all-time peak during January 2022. The RSV was on an upward trend since late 2021, coinciding with the discovery of the omicron variant. Interest in “COVID hair loss” during the second week of January 2022 was 14% higher than that during the mid-2020 peak of the initial COVID-19 surge, and 82% higher than that during the mid-2021 peak of the delta variant surge. While frequently cyclical, interest in “Telogen Effluvium” reached a new peak level of interest in December 2021, surpassing the mid-2020 peak by 19% and the mid-2021 peak by 33%. For each week between February 1, 2020, and January 16, 2022, the average weekly RSV for “COVID hair loss” was significantly associated with the number of new COVID-19 cases (*r*=0.59, Spearman rank correlation *P*<.001) (Figure 2). Regionally, search interest, derived as search term popularity as a proportion of total searches within an area, was generally greater for both “COVID hair loss” and “Telogen Effluvium” in higher-income countries including the United States and the United Kingdom, which demonstrated up to 10 times more interest than lower-income countries including South Africa and India. However, an outsized search interest was observed for “Telogen Effluvium” in the Philippines and Pakistan with an average of 12% higher search interest compared to the United States and the United Kingdom.

## Conclusions

People are paying more attention than ever before to COVID-19­–related hair loss and telogen effluvium, which may suggest a growing incidence of such cutaneous ailments in the COVID-19 pandemic environment. More directly, it is clear that the population believes in a linkage between hair loss symptoms and COVID-19. While increased interest was largely exhibited by higher-income countries, certain lower-income countries demonstrated similar interest levels, suggesting that this potential association is of a global nature and has widespread relevance. These findings align with those of other reports of diverse clinical scope, which suggest that associations between certain events, such as seasonal changes, and variation in Google search data for specific skin conditions may be indicative of health interests among the general public [5]. Though it is uncertain whether the heightened search interest in COVID-19 hair loss and its positive correlation with daily new COVID-19 cases stems from current or prior illness, breakthrough infectivity of the omicron variant, or greater media attention, the public is avidly searching for explanations. Dermatologists and other physicians will continue to be called upon to discuss this association in clinical practice, especially around periods of surging COVID-19 cases when internet search interest peaks.

## Abbreviations

RSV: relative search volume
